# Supplementary material for: Extracting multiple surfaces from 3D microscopy images in complex biological tissues with the Zellige software tool
Source: BMC Biol. 2022 Aug 23;20:183. doi: 10.1186/s12915-022-01378-0 (PMC9397159; doi:10.1186/s12915-022-01378-0)
Supplement: Supplementary file 1 — Additional file 1: Supplementary Notes 1-5. Note 1. Overview of Zellige implementation. Note 2. Generation of phantom 3D images. Note 3. Comparing the reconstructed and ground truth height maps. Note 4. Sensitivity Analysis. Note 5. Computational/processing time of Zellige. Table S1. Zellige parameters. [file 12915_2022_1378_MOESM1_ESM.zip › Additional_File_1_SupplementaryNotes_TableS1.pdf]

	Selection parameters					Construction parameters								
	T_A	T_{otsu}	S_{min}	σ_{XY}	σ_Z	T_{OSE1}	R_1	C_1	T_{OSE2}	R_2	C_2	**Surface**	**RMSE**	**Coverage**
**Phantom**	16	12	5	4	2	0.9	5	0.8	0.1	10	0.8	S1	0.23	100
												S2	0.31	100
												S3	0.56	100
**Fly**	6	1	5	5	1	0.7	10	0.8	0.1	5	0.9	S1	0.93	99
												S2	0.83	96
												S3	1.25	94
												S4	0.55	93
**Cochlea**	1	1	5	4	1	0.1	5	0.7	0.1	10	0.8	S1	1.08	100
**Culture**	23	16	5	4	1	0.9	5	0.9	0.1	5	0.8	S1	0.81	98
**Organoid**	5	12	5	2	1	0.9	5	0.8	0.1	10	0.8	S1	0.83	100
												S2	1.13	88
