# Supplementary figures and images for: Extracting multiple surfaces from 3D microscopy images in complex biological tissues with the Zellige software tool
Source: BMC Biol. 2022 Aug 23;20:183. doi: 10.1186/s12915-022-01378-0 (PMC9397159; doi:10.1186/s12915-022-01378-0)

**A****Pixel selection**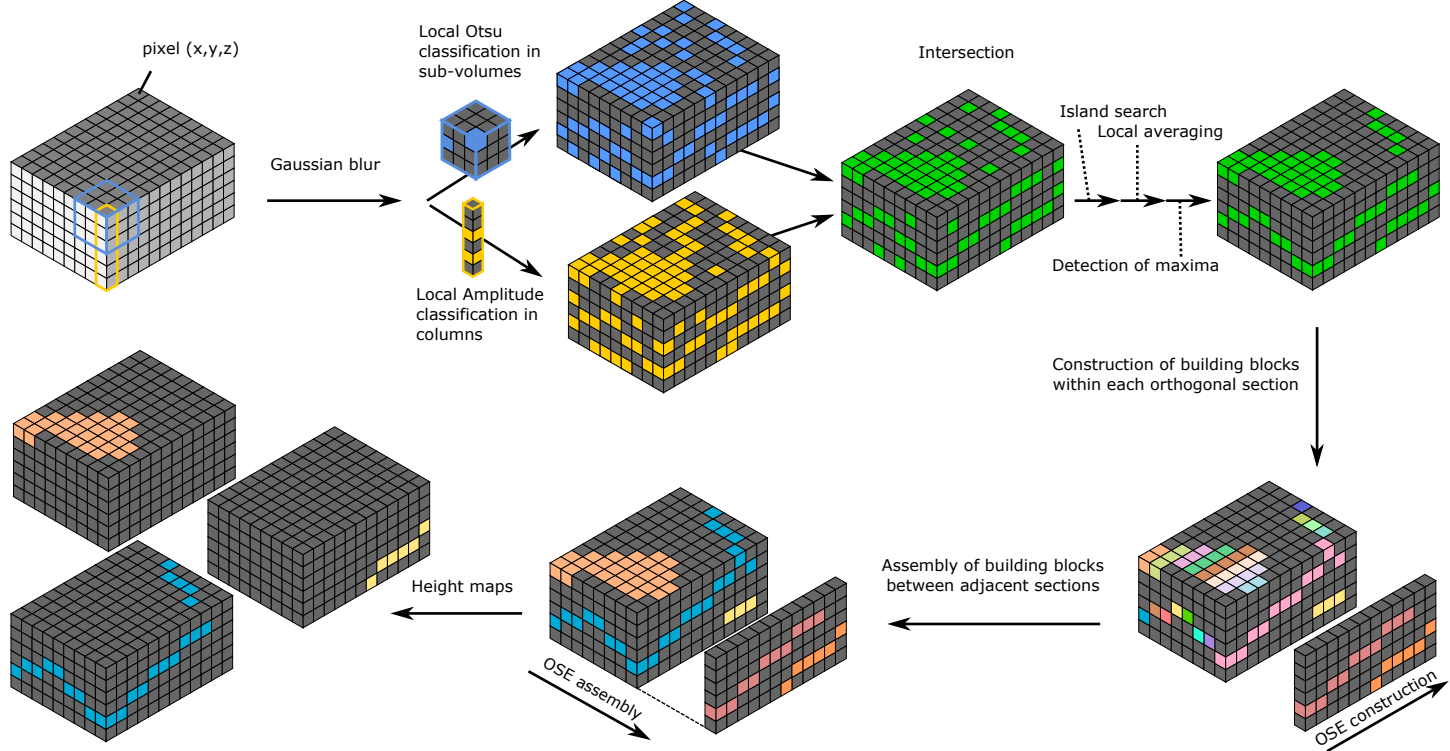**B**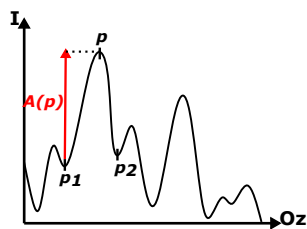**C**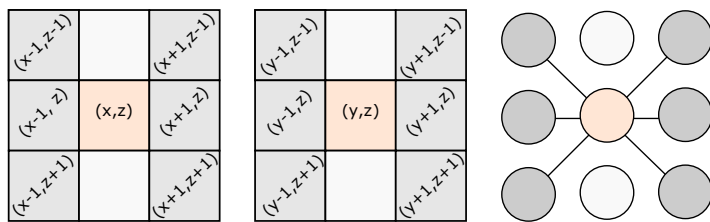**D**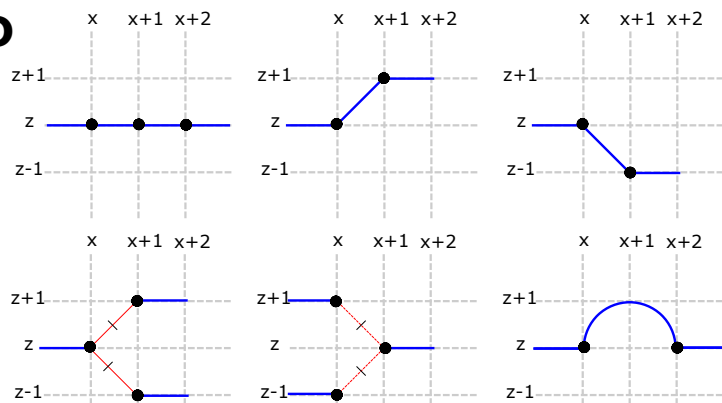**E**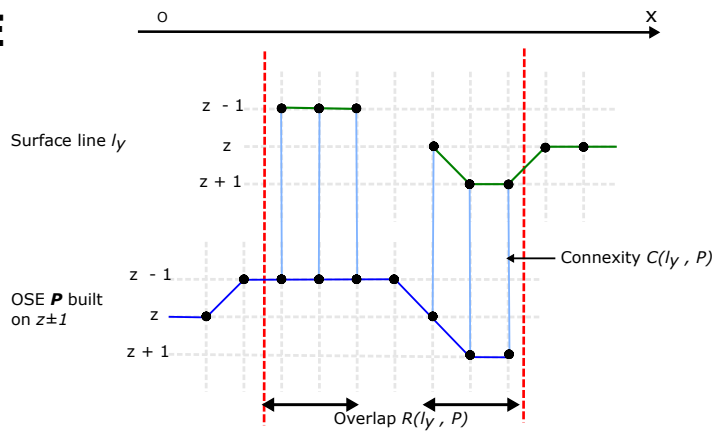

Supplement: Supplementary file 2 — Additional file 2: Figure S1. Zellige implementation. (A) The two main algorithmic steps of Zellige. Upper part: Surface voxel selection step (step 1). Lower part: Surface assembly step (step 2). (B) Determination of the amplitude A(p) of some local maximum p along the z-axis, relative to its two closest local minima p1 and p2. (C,D) Connectivity rules used to connect putative surface voxels together for the construction of orthogonal surface elements (OSEs). These rules are based on a 6-connectivity relationship defined within each orthogonal (xz or yz) section as shown in (C). This relationship is extended to allow the presence of single straight gaps, while being constrained to forbid the occurrence of forking points. The allowed local neighborhood configurations along an OSE are illustrated in (D) in the case of a construction within xz sections. The OSEs are formally defined as the connected components of the graph GOSE defined by these rules within each orthogonal section. (E) Compatibility rules used in the surface assembly step, illustrated here in the case where OSEs have been constructed within xz sections, and assembly proceeds along the y-axis. To validate the addition of a new OSE σ constructed within section y+1 (in blue) to the surface S under construction, whose intersection with section y is shown (surface line ly, in green), two quantities are computed: the overlap R(S,σ) is defined as the number of pixels of σ that share the x coordinate of some pixel of ly. The connectivity C(S,σ) is defined as the fraction of the overlapping pixels of σ that are 6-connected, in their respective yz section, to the corresponding point of ly (according to the 6-connectivity relationship shown in C, middle panel). In the depicted example, there are 2 OSEs (in green) with, for both of them, R(S,σ)=3 and C(S,σ)=3/3=1. The OSE will be added to S if R(S,σ) ≥ R0 and C(S,σ) ≥ C0, where R0 and C0 are tunable thresholds that set the stringency of the matching condition. [file 12915_2022_1378_MOESM2_ESM.pdf]

# A

## Selection Parameters

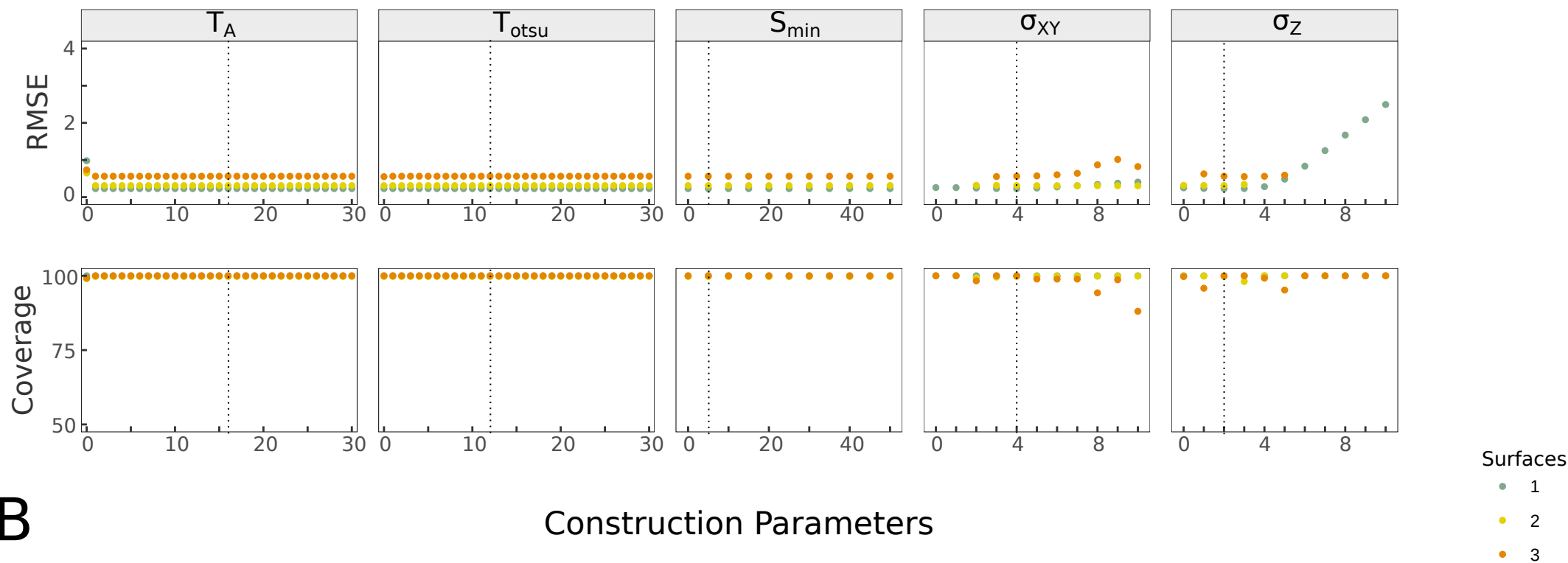

# B

## Construction Parameters

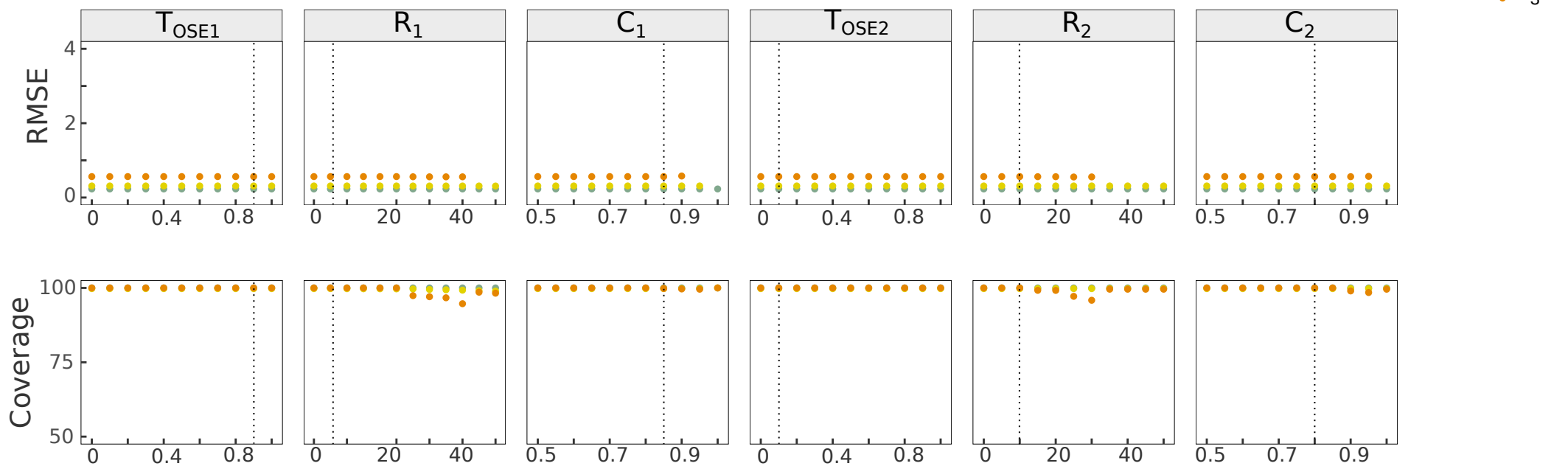

Supplement: Supplementary file 3 — Additional file 3: Figure S2. Sensitivity analysis of Zellige on the phantom image. (A) Surface voxel selection parameters. (B) Surface assembly parameters. Reference values are indicated by the dashed line. [file 12915_2022_1378_MOESM3_ESM.pdf]

# A

## Selection Parameters

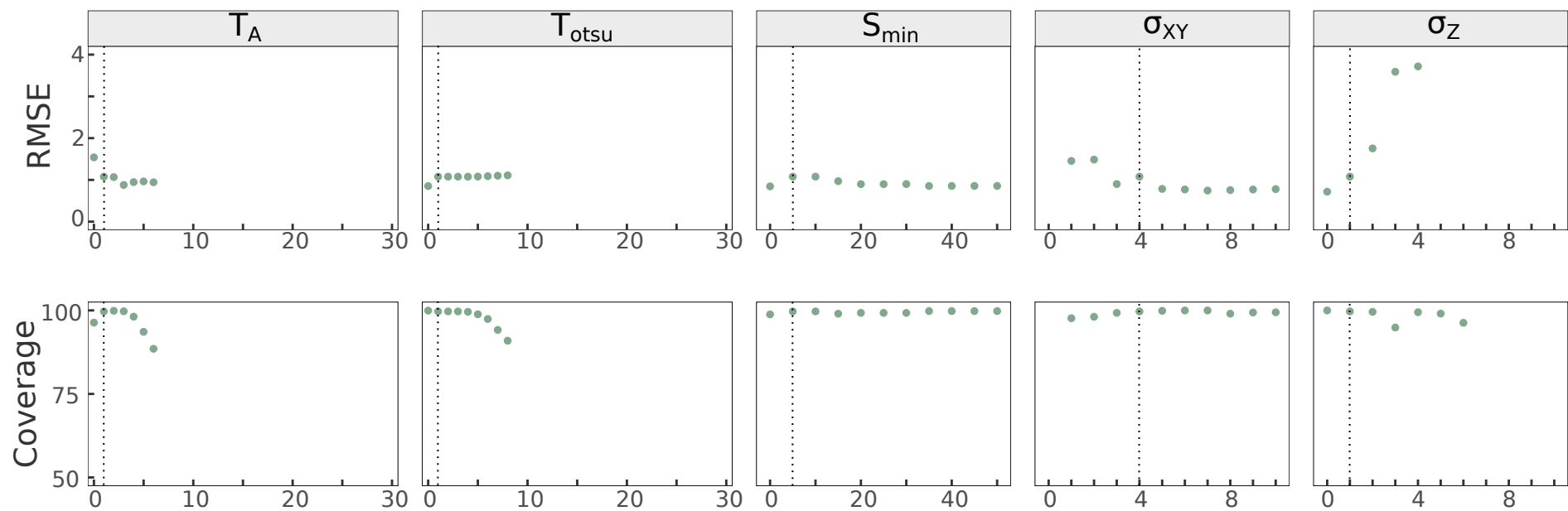

# B

## Construction Parameters

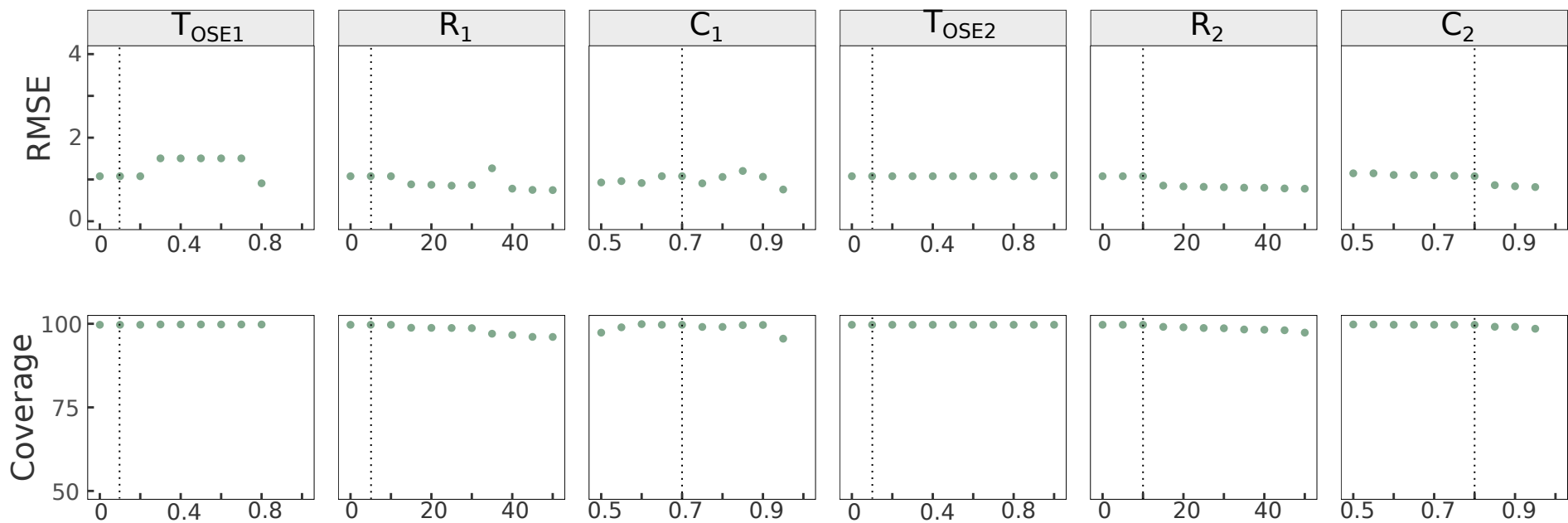

Supplement: Supplementary file 5 — Additional file 5: Figure S4. Sensitivity analysis of Zellige on the cochlear epithelium image. (A) Surface voxel selection parameters. (B) Surface assembly parameters. Reference values are indicated by the dashed line. [file 12915_2022_1378_MOESM5_ESM.pdf]

**A**

## Selection Parameters

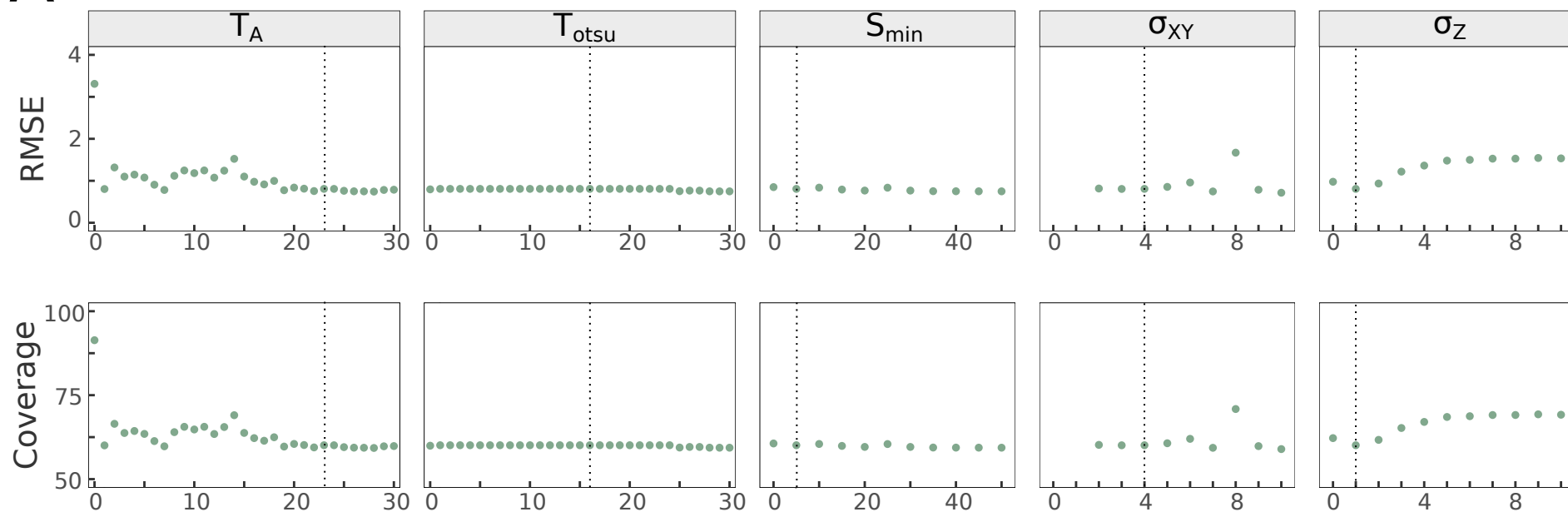**B**

## Construction Parameters

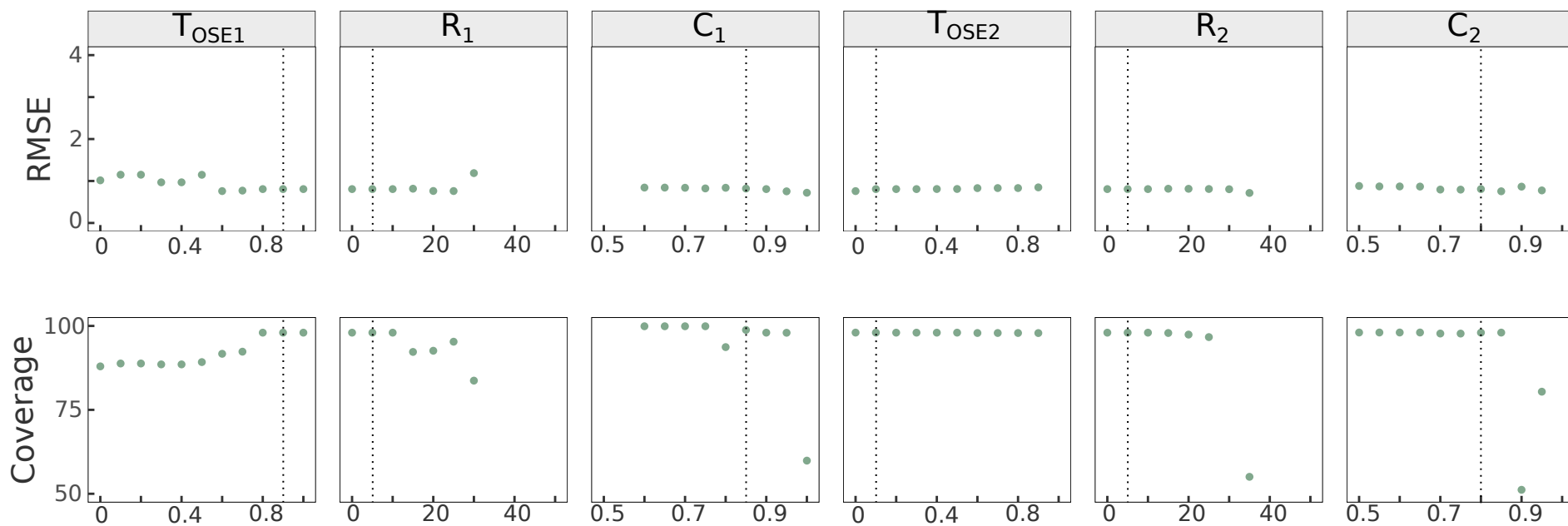

Supplement: Supplementary file 6 — Additional file 6: Figure S5. Sensitivity analysis of Zellige on the primary epithelium culture image. (A) Surface voxel selection parameters. (B) Surface assembly parameters. Reference values are indicated by the dashed line. [file 12915_2022_1378_MOESM6_ESM.pdf]

**A**

## Selection Parameters

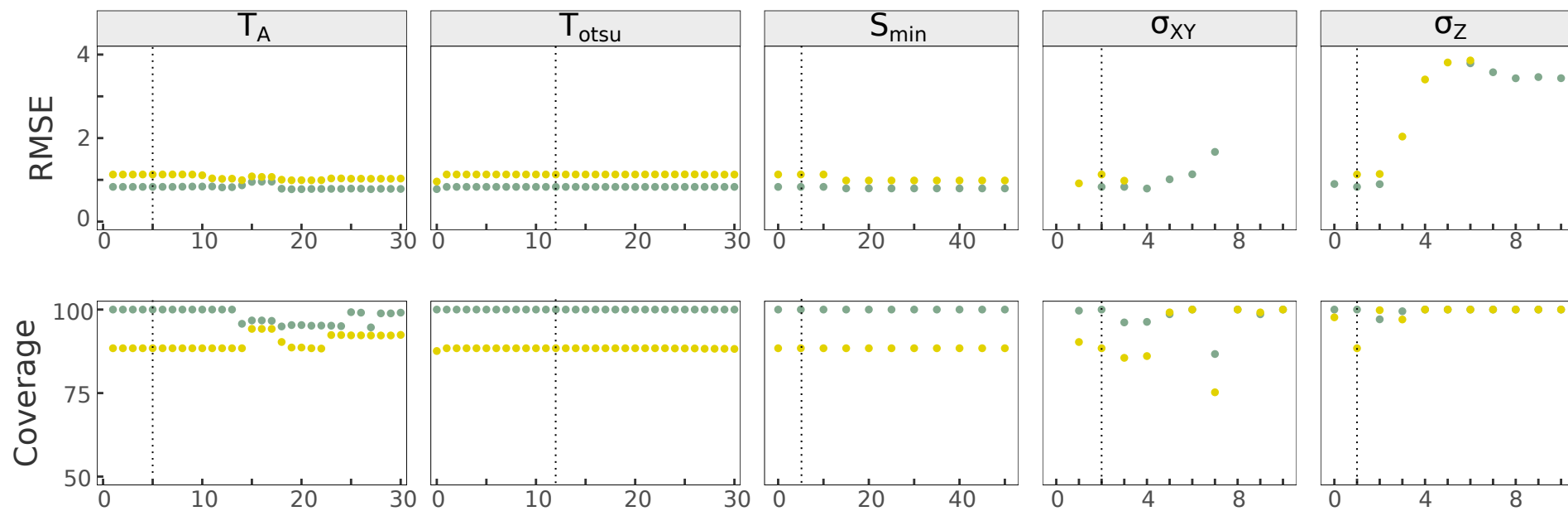**B**

## Construction Parameters

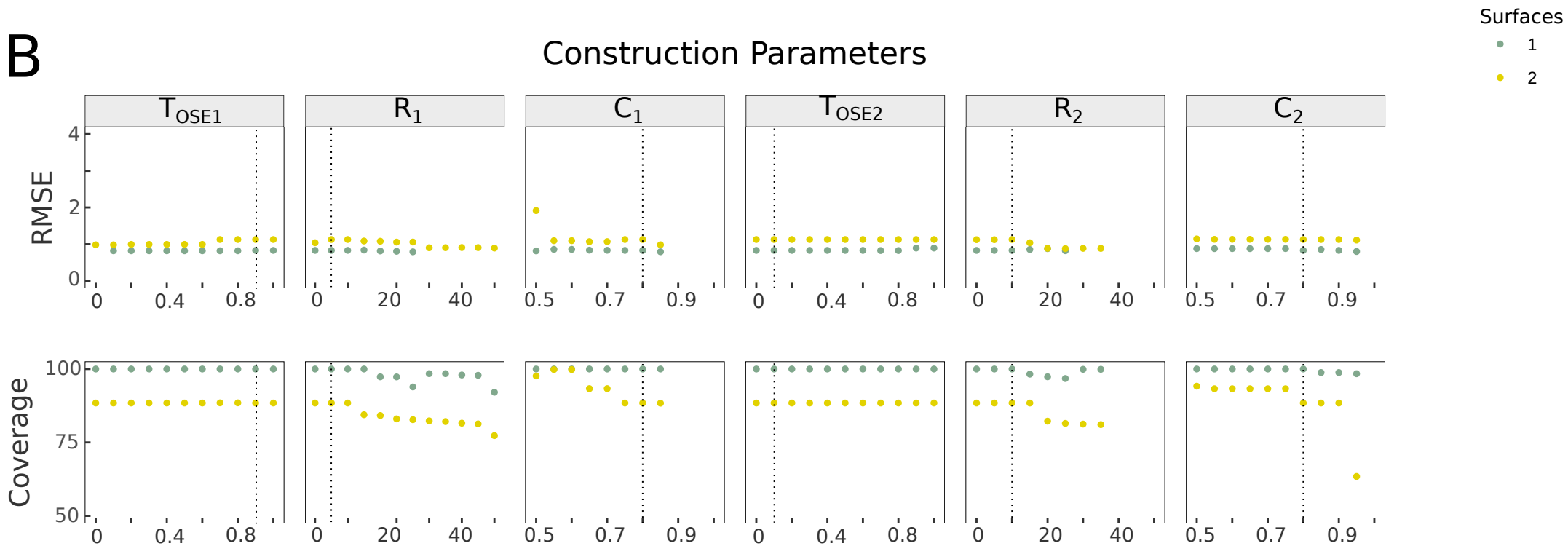

Surfaces

1

2

Supplement: Supplementary file 7 — Additional file 7: Figure S6. Sensitivity analysis of Zellige on the inner ear organoid image. (A) Surface voxel selection parameters. (B) Surface assembly parameters. Reference values are indicated by the dashed line. [file 12915_2022_1378_MOESM7_ESM.pdf]

A

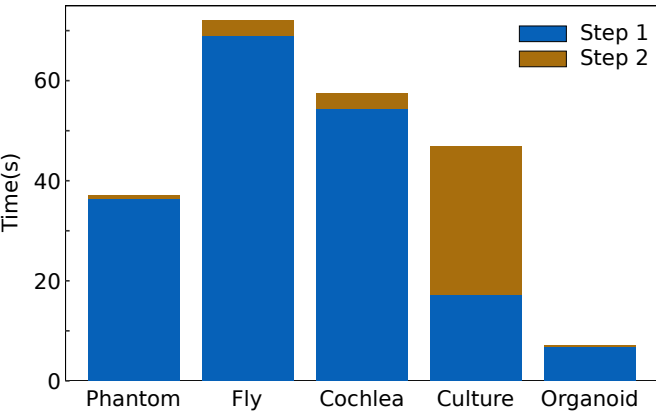

B

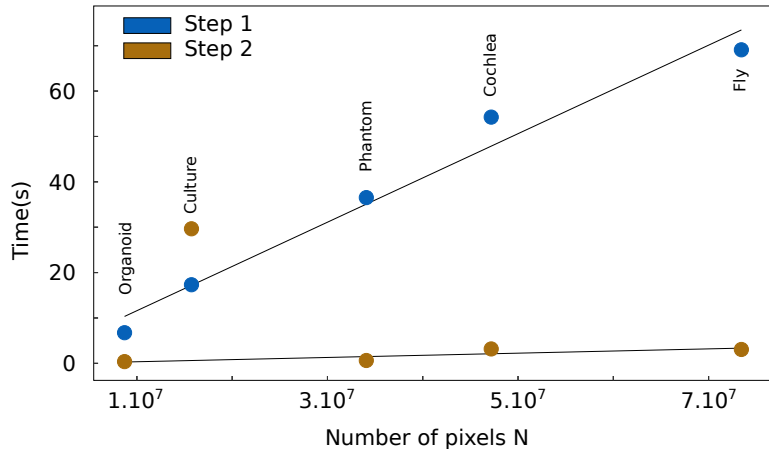

Supplement: Supplementary file 8 — Additional file 8: Figure S7. Computational time analysis of Zellige. (A) Computation times (step 1 in blue, step 2 in brown) for processing the various images tested on a PC notebook computer with (processor Intel Core i9 2,4 GHz with 32 Gb of Ram). Apart for the case of a highly rough surface (culture specimen) the computation time is largely dominated by the surface voxel selection step (step 1). (B) The same computation times are re-plotted as a function of image size N (number of voxels). Note the linear growth of the computational time of step 1 as a function of N, while that of step 2 shows much slower growth. [file 12915_2022_1378_MOESM8_ESM.pdf]
