# Supplementary material for: Extracting multiple surfaces from 3D microscopy images in complex biological tissues with the Zellige software tool
Source: BMC Biol. 2022 Aug 23;20:183. doi: 10.1186/s12915-022-01378-0 (PMC9397159; doi:10.1186/s12915-022-01378-0)

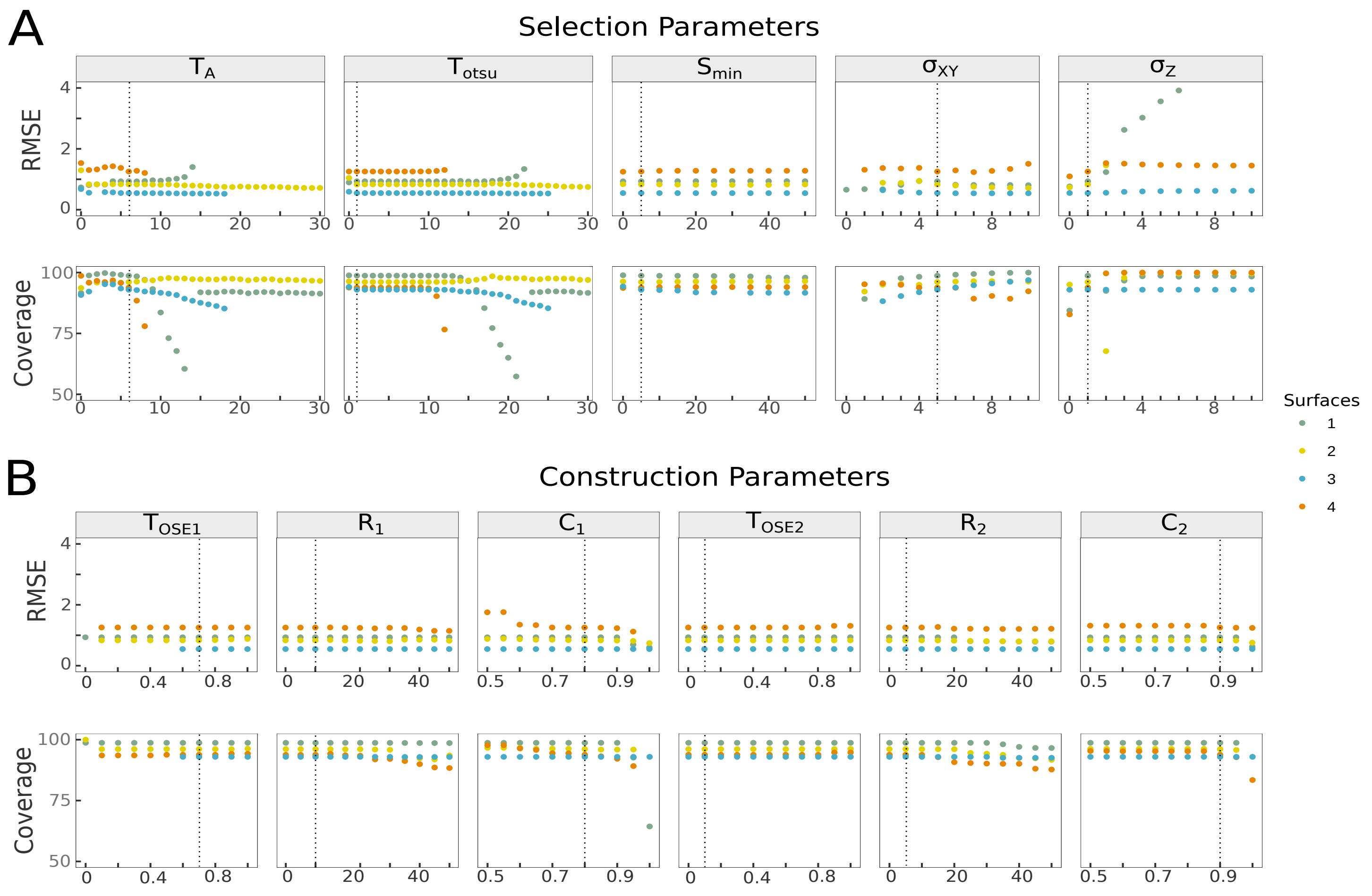

C

	$\sigma_{XY} = 1$		$\sigma_{XY} = 3$		$\sigma_{XY} = 5$		$\sigma_{XY} = 9$		$\sigma_{XY} = 10$	
	RMSE	Coverage	RMSE	Coverage	RMSE	Coverage	RMSE	Coverage	RMSE	Coverage
S1	0,68	89	0,81	98	0,93	99	0,81	100	0,80	100
S2	NA	NA	0,87	96	0,84	96	0,72	96	0,71	96
S3	1,31	85	1,35	95	1,25	94	1,34	89	1,51	92
S4	NA	NA	0,59	90	0,55	93	0,54	96	0,54	97

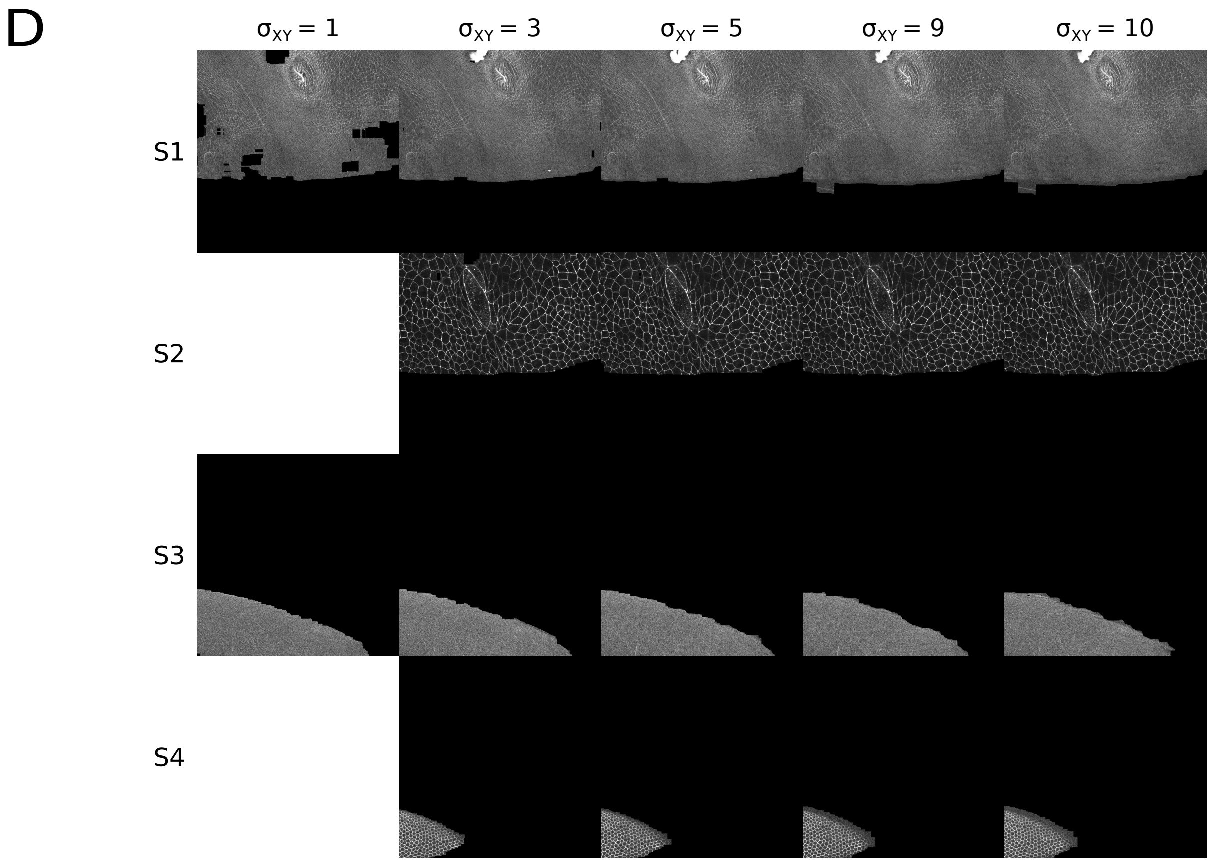

Supplement: Supplementary file 4 — Additional file 4: Figure S3. Sensitivity analysis of Zellige on the pupal fly image. (A) Surface voxel selection parameters. (B) Surface assembly parameters. Reference values are indicated by the dashed line. (C) RMSE and coverage results obtained for different values of the σxy blur taken within and outside the interval defined by the parameter sweep in (A, “σxy” panel). (D) Visualization of the projections on the extracted surfaces obtained for the different σxy blur values shown in (C). [file 12915_2022_1378_MOESM4_ESM.pdf]
